# Development and validation of a nomogram for assessing risk of isolated high 2-hour plasma glucose

**DOI:** 10.3389/fendo.2022.943750

**Published:** 2022-09-08

**Authors:** Kan Sun, Xianchao Xiao, Lili You, Xiaosi Hong, Diaozhu Lin, Yujia Liu, Chulin Huang, Gang Wang, Feng Li, Chenglin Sun, Chaogang Chen, Jiahui Lu, Yiqin Qi, Chuan Wang, Yan Li, Mingtong Xu, Meng Ren, Chuan Yang, Guixia Wang, Li Yan

**Affiliations:** ^1^ Department of Endocrinology, Sun Yat-sen Memorial Hospital, Sun Yat-sen University, Guangzhou, China; ^2^ Department of Endocrinology, The First Hospital of Jilin University, Changchun, China; ^3^ Department of Clinical Nutrition, Sun Yat-sen Memorial Hospital, Sun Yat-sen University, Guangzhou, China

**Keywords:** diabetes mellitus, hyperglycemia, 2h OGTT, nomogram model, risk assessment model

## Abstract

A tool was constructed to assess need of an oral glucose tolerance test (OGTT) in patients whose fasting plasma glucose (FPG) and hemoglobin A1c (HbA1c) are normal. Data was collected from the longitudinal REACTION study conducted from June to November 2011 (14,686 subjects, aged ≥ 40 y). In people without a prior history of diabetes, isolated high 2-hour plasma glucose was defined as 2-hour plasma glucose ≥ 11.1 mmol/L, FPG < 7.0 mmol/L, and HbA1c < 6.5%. A predictive nomogram for high 2-hour plasma glucose was developed *via* stepwise logistic regression. Discrimination and calibration of the nomogram were evaluated by the area under the receiver operating characteristic curve (AUC) and Hosmer-Lemeshow test; performance was externally validated in Northeast China. Parameters in the model included gender, age, drinking status, marriage status, history of hypertension and hyperlipidemia, waist-to-hip ratio, FPG, and HbA1c. All variables were noninvasive, except FPG and HbA1c. The AUC of the nomogram for isolated high 2-hour plasma glucose was 0.759 (0.727-0.791) in the development dataset. The AUCs of the internal and externally validation datasets were 0.781 (0.712-0.833) and 0.803 (0.778-0.829), respectively. Application of the nomogram during the validation study showed good calibration, and the decision curve analysis indicated that the nomogram was clinically useful. This practical nomogram model may be a reliable screening tool to detect isolated high 2-hour plasma glucose for individualized assessment in patients with normal FPG and HbA1c. It should simplify clinical practice, and help clinicians in decision-making.

## Introduction

Diabetes mellitus (DM) is a metabolic disorder that impairs biological function. The etiological factors are both genetic and environmental. In 2017, it was estimated that 9.7% of the United States population had DM (1). In the same year, the prevalence of DM in China was 12.8% (2). The high prevalence of DM and its related disability and mortality has made it a critical health problem worldwide (3–5). Delays in the diagnosis and treatment of DM lead to a greater incidence of associated mortality. An improved method for identifying hyperglycemia could significantly benefit the population at risk (6–8).

A diagnosis of DM is currently based on plasma glucose criteria: fasting plasma glucose (FPG); the 2-hour oral glucose tolerance test (2-h OGTT); or hemoglobin A1c (HbA1c) (9). Each is considered standard, but measuring FPG and HbA1c levels is more convenient than administering the 2-h OGTT. The latter requires two blood samples, and is time-consuming and cumbersome for both the primary care physician and the patients. Especially when both the FPG and HbA1c levels are normal, compliance with the 2-h OGTT may be neglected (10). A simple and convenient tool is urgently needed, that is appropriately sensitive and specific, to assess the risk of OGTT ≥ 11.1 mmol/L at 2 hours (i.e., isolated high 2-hour plasma glucose), in populations with FPG and HbA1c levels that are within normal range.

There have been several reports of assessment models that identify risk of DM, with areas under the receiver-operating characteristic curve (AUC) of 0.60 to 0.80. The majority of these models outperformed their validation datasets in their original population (11–13). A nomogram model designed to assess risk factors for DM based on 8999 patients in Korea showed an AUC of ~0.80 (14). In 2013, Zhou et al. (12) developed the new Chinese Diabetes Risk Score for detecting DM in a Chinese population, and the AUC for undiagnosed type 2 diabetes was 0.748. Most mathematical models designed for assessing risk of DM have not included 2-h OGTT, and their accuracy has been low.

The 2-h OGTT has been the mainstay for diagnosing DM for decades, and is the gold standard recognized by the American Diabetes Association (9). Two-hour OGTT detects diabetes most efficiently and provides metabolically relevant information. Furthermore, ~40% of subjects who later develop DM are within the normal glucose tolerance at OGTT. These subjects constitute a large reservoir of future DM cases (15). Yet, there is no nomogram to depict an individual’s probability of DM based on isolated high 2-hour plasma glucose.

The present study initially developed and validated a convenient predictive nomogram for patients with FPG and HbA1c levels within normal range, who require a 2-h OGTT. This approach should improve our ability to identify individuals who are at high risk of isolated high 2-hour plasma glucose, and facilitate a more personalized approach to their care.

## Methods

### Participants

From June to November of 2011, a study known as REACTION (i.e., Risk Evaluation of cAncers in Chinese diabeTic Individuals: a lONgitudinal) was performed in China. REACTION was a multi-institutional large prospective cohort study. REACTION was conducted in 25 localities across the country, divided by geographic location (Northeast, North, East, South Central, Northwest, and Southwest China). Participants in the research had to be at least 40 years old and were identified using local registration data. The results of this research have already been published (16, 17). The study procedure followed the principles of the Helsinki Declaration II and was approved by the Ethics Committee of Ruijin Hospital (IRB number: [2011] Ethics Record No (14).

**Figure 1:**
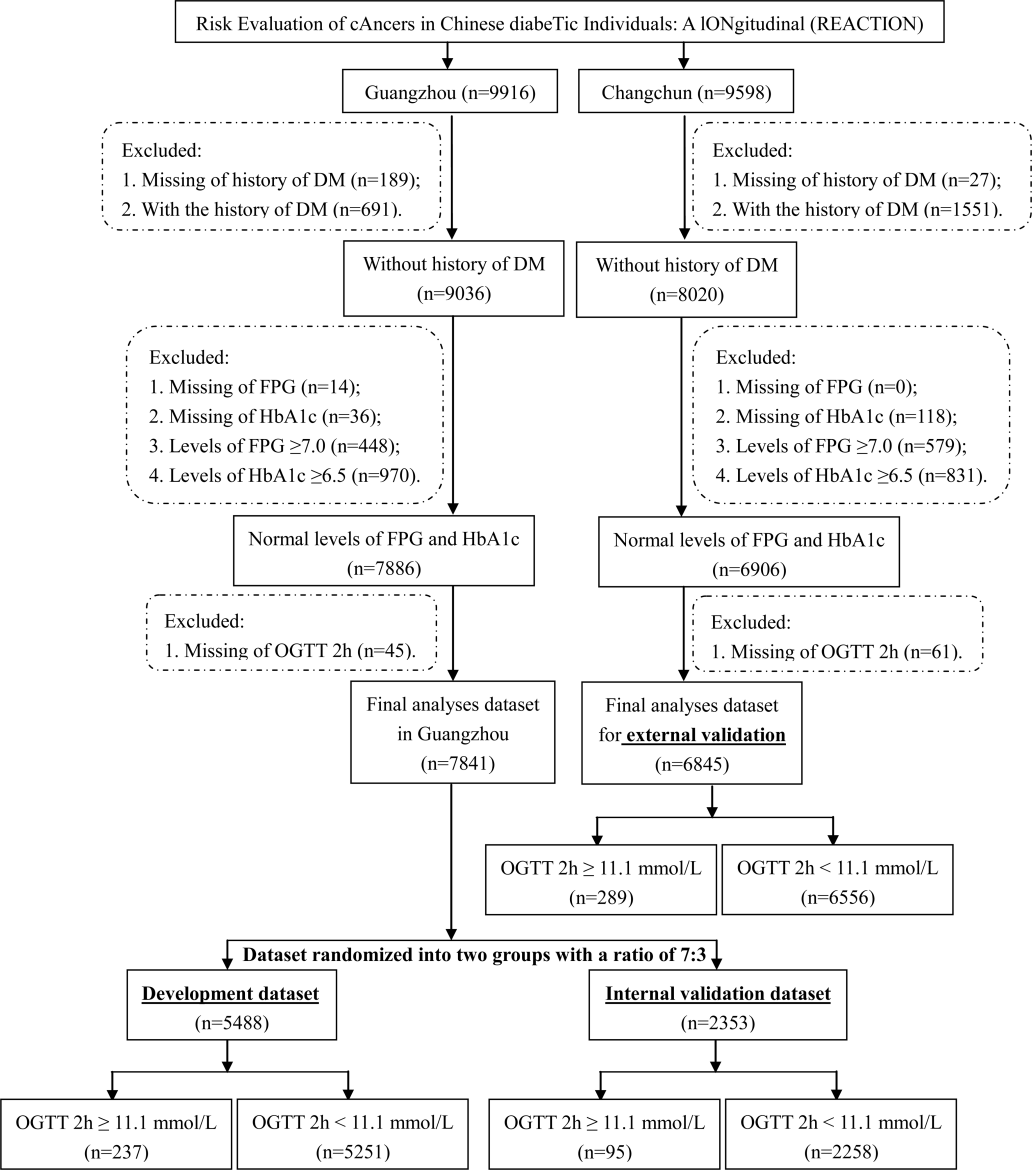
Flow chart of the selection of the research participants.

To construct the assessment model, data for the development and internal validation populations were collected in Guangzhou, South China (**Figure 1**), which was cross-sectional data and one of the parts of the REACTION study. During the recruitment process, 10,104 residents aged 40 and above were requested to participate by examination notifications or home visits, and 9916 (98.1%) signed the consent form and consented to take part in the survey.

For the external validation of the assessment model, data for the external validation population was collected from Changchun, Northeast China (**Figure 1**), which was cross-sectional data and another parts of REACTION study. Of the 10,080 residents aged at least 40 years, 9598 (95.2%) signed the consent form and completed the survey.

Participants in the initial development and validation populations were excluded for the following reasons: history of DM or lack of information regarding history of DM; lack of FPG or HbA1c data; FPG ≥ 7.0 mmol/L or HbA1c ≥ 6.5%; or missing 2-hour OGTT data.

Finally, 7841 and 6845 individuals were included in the development and validation groups, from Guangzhou and Changchun, respectively (**Figure 1**). The missing-at-random assumption was used for missing data, and multiple imputations were done using the multivariate imputation by chained equations (MICE) approach with 5 imputed datasets and 10 iterations (18–20).

### Clinical and biochemical measurements

A standardized data form was created to retrieve all relevant information on lifestyle factors, medical histories, socio demographics, and family histories. Information collected by trained medical personal included the following: gender; age; family history of DM (yes or no); marriage status (married or cohabitating, unmarried, and others); history of hypertension (yes or no); and hyperlipidemia (yes or no). Never, current (smoking or drinking frequently over the preceding 6 months), or ever were the categories for smoking and drinking behaviors (prior to the previous 6 months only). By adding questions on the frequency and duration of walking and moderate or vigorous activities, a condensed version of the International Physical Activity Questionnaire (IPAQ) was utilized to evaluate physical activity during leisure time (21). For evaluating overall physical activity, separate metabolic equivalent hours per week (MET-h/week) calculations were made.

All subjects had their anthropometric measures taken using conventional methods by well-trained assessors. With individuals clothed in light indoor clothing and no shoes, body weight and standing height were measured to the closest 0.1 cm and 0.1 kg, respectively. The Body Mass Index (BMI) was computed by dividing the weight in kilograms by the height in meters squared (kg/m^2^). At the end of a mild expiration, participants’ waist circumference (WC) was measured at the umbilical level to the closest 0.1 cm while standing. A plastic flexible tape was used to measure the hipline above the great trochanters to the closest 0.1 cm. The waist-to-hip ratio (WHR) was computed by subtracting the WC from the hipline. After the participants had been sitting and resting peacefully for more than 5 minutes with feet on the ground and back supported, blood pressure measurements were taken three times consecutively by the same observer using an automated electronic instrument (OMRON, Omron, China). For the analysis, each subject’s mean systolic and diastolic blood pressures (SBP and DBP) were employed.

Participants were requested to fast for at least 10 hours before the baseline survey, and venous blood samples were obtained for laboratory tests by competent nurses. Blood samples were centrifuged at 2500g for 15 minutes to extract serum or plasma within 2 hours of collection and kept at –80°C at the Shanghai Institute of Endocrine and Metabolic Disease’s Central Laboratory (certified by the College of American Pathologists). For both the Guangzhou and Changchun datasets, HbA1c was measured using high-performance liquid chromatography (Bio-Rad, Hercules, CA, USA) at the Central Laboratory.

Individuals were required to attend a clinical facility where a health care professional can obtain venous blood samples at times 0 and 2 h after an oral 75-g anhydrous glucose challenge. For the Guangzhou dataset, measurements of FPG and 2-h plasma glucose were performed using an autoanalyzer (Beckman CX-7 Biochemical Auto-analyzer, Brea, CA) in the Central Laboratory of Sun Yat-sen Memorial Hospital. For the Changchun dataset, these measurements were conducted with a Bayer ADVIA2400 (Leverkusen, Germany) in the Central Laboratory of First Hospital of Jilin University.

### Development of an individualized assessment model

Using a simple random sample procedure, the 7841 subjects registered in Guangzhou were separated into development model (n = 5488) and internal validation (n = 2353) datasets in a 7:3 ratio. Recognition models for undiagnosed isolated high 2-hour plasma glucose were developed using data from the training set. The assessment model was created using a multivariable binary logistic regression model and the backward stepwise selection approach.

Gender, age, drinking status, married status, history of hypertension, history of hyperlipidemia, SBP, DBP, BMI, WC and WHR were all included as noninvasive clinical variables in multivariable logistic regression analysis. The likelihood ratio test using Akaike’s information criteria was used to do backward stepwise selection (AIC). The final model was chosen based on its lowest AIC.

The noninvasive model included the following factors: gender, age, drinking status, marriage status, history hypertension, history of hyperlipidemia, SBP and WHR. The FPG and HbA1c results were added to the noninvasive model and considered for final construction of the nomogram. For a quantitative tool to assess the individual probability of isolated high 2-hour plasma glucose, a nomogram was established for further application using the rms package in RStudio software version 3.6.1.

### The nomogram’s validation and calibration

The internal and external validation populations (Guangzhou, n = 2353 and Changchun, n = 6845, respectively) were used to validate the nomogram model from the development population. The accuracy of the models was assessed using a receiver operating characteristic (ROC) curve analysis. The concordance index was used to measure the model’s effectiveness in assessing isolated high 2-hour plasma glucose (C-index). The calibration of the nomogram was assessed using a calibration plot and the Hosmer-Lemeshow test. Using rmda in RStudio Version 3.6.1, decision curve analysis was used to evaluate the net benefit of the nomogram models at various threshold probabilities in the datasets.

### Statistical methods

Except for skewed variables, which are given as median, continuous variables are shown as mean standard deviation (interquartile range, IQR). In both the Guangzhou and Changchun datasets, an independent samples t-test or a Kruskal-Wallis H test was employed to analyze the differences between subjects with and without isolated high 2-hour plasma glucose. Numbers are used to express categorical variables (percent). The differences based on isolated high 2-hour plasma glucose were compared using Chi-squared testing. The impact of clinical and biochemical measures on the prevalence of isolated high 2-hour plasma glucose was determined using logistic regression, and the results were displayed with odds ratios (ORs) and 95 percent confidence intervals (95% CIs). Model 1 included noninvasive clinical factors selected after backward stepwise regression with AIC in multivariate logistic regression. Model 2 further included FPG and HbA1c based on Model 1.

RStudio program was used for statistical analysis (version 3.6.1). All statistical tests were two-sided, with a significance level of 0.05.

## Results

### Clinical characteristics

After excluding participants with FPG ≥ 7.0 mmol/L or HbA1c ≥ 6.5%, the prevalence of isolated high 2-hour plasma glucose was 4.11% in Guangzhou and 4.22% in Changchun, and the two datasets were similar (**Table 1**; *P* = 1.000). Participants with isolated high 2-hour plasma glucose were significantly older; had greater BMI and WC; and higher FPG, HbA1c, and blood pressure; compared with those without isolated high 2-hour plasma glucose. In both regions (Guangzhou and Changchun), the participants with isolated high 2-hour plasma glucose were significantly more likely to have a history of hyperlipidemia, hypertension and metabolic syndrome.

**Table 1:** Characteristics of participants in the Guangzhou and Changchun datasets in China; without and with isolated high 2-hour plasma glucose.

	Guangzhou			Changchun		
	No	Yes*	*P*	No	Yes*	*P*
Subjects, n	7509	332		6556	289	
Age, y	55.16 ± 7.64	58.62 ± 9.89	<0.001	56.85 ± 9.77	61.36 ± 10.17	<0.001
Male	2099 (27.95)	109 (32.83)	0.0613	2070 (31.57)	118 (40.83)	0.001
Family history of DM	1140 (15.19)	59 (17.78)	0.228	754 (11.50)	33 (11.42)	1.000
Current smoking	745 (9.92)	40 (12.05)	0.416	908 (13.85)	45 (15.57)	0.703
Current drinking	246 (3.28)	22 (6.63)	0.003	557 (8.50)	38 (13.15)	0.018
Married or cohabitating	6801 (90.57)	283 (85.24)	0.005	6051 (92.30)	267 (92.39)	0.709
Hypertension history	997 (13.28)	86 (25.90)	<0.001	1051 (16.03)	90 (31.14)	<0.001
Hyperlipidemia history	461 (6.14)	41 (12.35)	<0.001	422 (6.44)	34 (11.76)	<0.001
SBP, mmHg	124.11 ± 15.27	131.40 ± 16.56	<0.001	138.43 ± 21.53	148.85 ± 21.87	<0.001
DBP, mmHg	74.77 ± 9.51	77.16 ± 9.79	<0.001	79.95 ± 11.94	82.91 ± 11.77	<0.001
Height, cm	158.39 ± 7.40	157.74 ± 7.07	0.103	161.43 ± 7.58	161.01 ± 7.67	0.366
Weight, kg	58.56 ± 9.11	59.00 ± 9.25	0.395	64.69 ± 10.78	66.35 ± 11.31	0.015
BMI, kg/m^2^	23.29 ± 2.96	23.68 ± 3.19	0.033	24.76 ± 3.29	25.53 ± 3.59	<0.001
WC, cm	80.59 ± 8.72	82.49 ± 9.46	<0.001	83.01 ± 9.26	86.17 ± 8.23	<0.001
Hipline, cm	93.60 ± 6.50	93.57 ± 6.98	0.933	96.91 ± 6.92	98.47 ± 6.73	<0.001
WHR	0.86 ± 0.06	0.88 ± 0.06	<0.001	0.86 ± 0.06	0.87 ± 0.05	<0.001
FPG, mmol/L	5.13 ± 0.57	5.78 ± 0.65	<0.001	5.47 ± 0.53	5.98 ± 0.56	<0.001
HbA1c,%	5.79 ± 0.35	5.97 ± 0.32	<0.001	5.67 ± 0.38	5.94 ± 0.35	<0.001
Physical activity (MET-h/week)	21.0[10.5, 45.0]	21.0[9.0, 43.0]	0.185	21.0[0.0, 42.0]	20.0[0.0, 31.5]	0.371
Prevalence of metabolic syndrome (%)	1120 (16.25)	101 (30.42)	<0.001	1731 (26.40)	152 (52.60)	<0.001
Prevalence of hypertension (%)	1843 (24.54)	146 (43.98)	<0.001	3122 (47.62)	198 (68.51)	<0.001
Prevalence of dyslipidemia (%)	5392 (71.81)	269 (81.02)	<0.001	4225 (64.44)	217 (75.09)	<0.001

*Without (no) and with (yes) isolated high 2-hour plasma glucose. Guangzhou and Changchun datasets are n = 7841 and n = 6845, respectively. Data are reported as n (%) for categorical variables and mean ± SD or median (interquartile ranges) for skewed variables. The P-value is derived from the univariable association analyses between each of the characteristics and isolated high 2-hour plasma glucose.

Associations between isolated high 2-hour plasma glucose and the selected biochemical measurements were confirmed by the logistic regression analyses (**Table 2**). Participants with the following were more likely to show high 2-hour plasma glucose: drinking (OR: 1.04, 95% CI: 0.79-1.35); non-married (OR: 1.15, 95% CI: 0.92-1.42); and with a history of hypertension (OR: 1.48, 95% CI: 1.06-2.06). Moreover, the following were significantly associated with an increased prevalence of isolated high 2-hour plasma glucose: SBP (OR: 1.01, 95% CI: 1.00-1.02); FPG (OR: 2.72, 95% CI: 2.16-3.43); and HbA1c (OR: 3.43, 95% CI: 2.21-5.39).

**Table 2:** Risk of factors for isolated high 2-hour plasma glucose in the development dataset ^1^.

	Model 1			Model 2		
	β	OR (95% CI)	*P*	β	OR (95% CI)	*P*
Intercept	-10.178	—	<0.001	-20.543	—	<0.001
Age	0.029	1.03 (1.01, 1.05)	<0.001	0.020	1.02 (1.00, 1.04)	0.020
Female	0.051	1.05 (0.78, 1.44)	0.745	0.049	1.05 (0.77, 1.44)	0.757
Drinking status	0.056	1.06 (0.81, 1.36)	0.669	0.041	1.04 (0.79, 1.35)	0.762
Marriage status	0.134	1.15 (0.92, 1.41)	0.203	0.139	1.15 (0.92, 1.42)	0.209
Hypertension history	0.410	1.51 (1.08, 2.07)	0.013	0.395	1.48 (1.06, 2.06)	0.019
Hyperlipidemia history	0.331	1.39 (0.90, 2.07)	0.117	0.271	1.31 (0.84, 1.97)	0.210
SBP	0.018	1.02 (1.01, 1.03)	<0.001	0.012	1.01 (1.00, 1.02)	0.011
WHR^2^	0.032	1.03 (1.01, 1.05)	0.004	0.020	1.02 (1.00, 1.04)	0.072
Physical activity (MET-h/week)	-0.002	1.00 (0.99, 1.00)	0.240	-0.003	1.00 (0.99, 1.00)	0.114
FPG	NA	NA	NA	1.001	2.72 (2.16, 3.43)	<0.001
HbA1c	NA	NA	NA	1.232	3.43 (2.21, 5.39)	<0.001
C-index datasets						
Development		0.676 (0.641-0.711)			0.759 (0.727-0.791)	
Internal validation		0.664 (0.602-0.726)			0.773 (0.712-0.833)	
External validation		0.686 (0.655-0.716)			0.803 (0.778-0.829)	

^1^β is the regression coefficient. Participant without (with) isolated high 2-hour plasma glucose was defined as 0 (1).

^2^Increasing for 0.01 units. NA, Not Available.

### Development and validation of a predictive model for 2-h OGTT glucose ≥ 11.1 mmol/L

**Figure 2:**
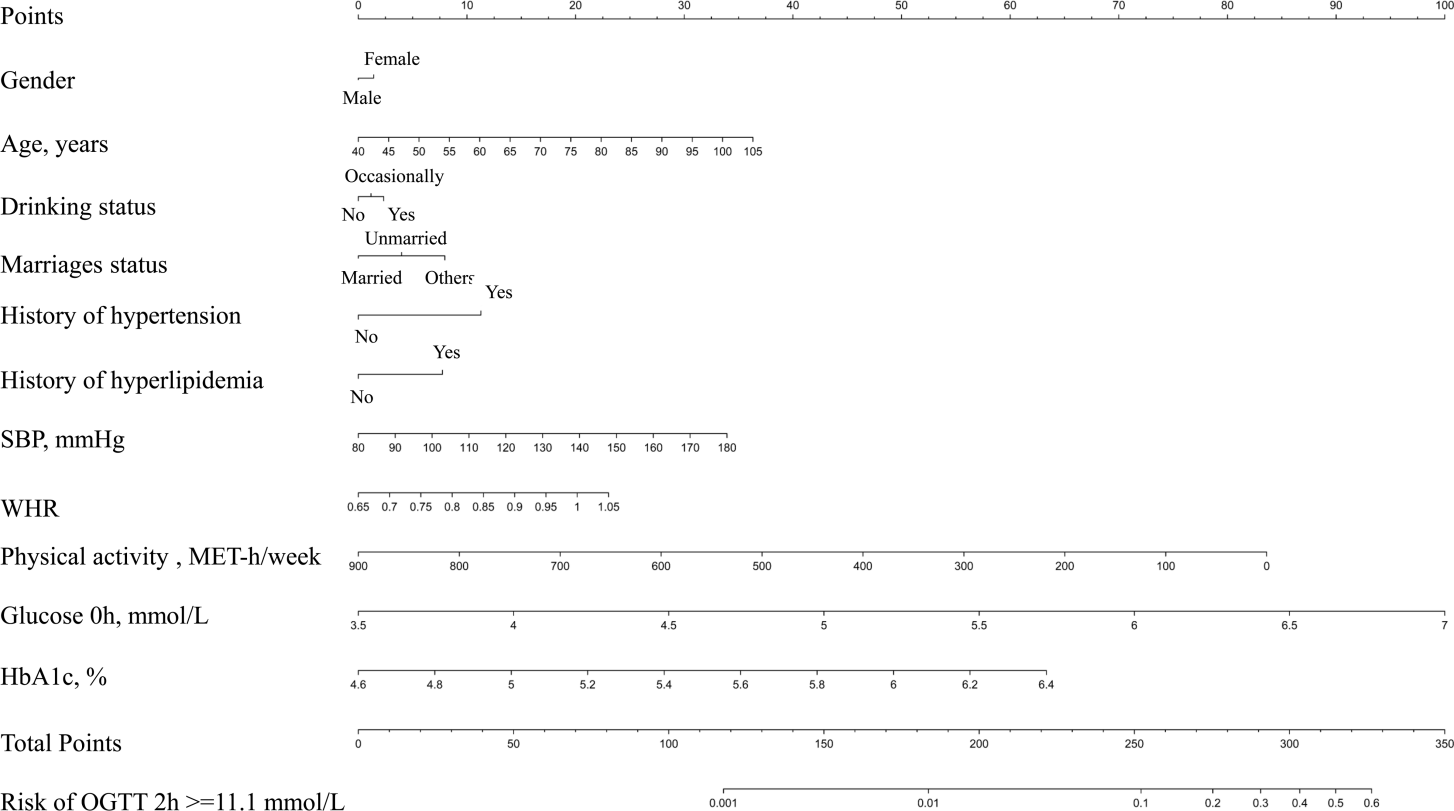
Nomogram for participants with isolated high 2-hour plasma glucose.

The best noninvasive model (model 1) generated after backward stepwise regression with AIC in multivariate logistic regression suggested that the following might be factors that influence the incidence of isolated high 2-hour plasma glucose: gender, age, drinking status, marriage status, history of hypertension, history of hyperlipidemia, SBP, and WHR. After adding FPG and HbA1c to the noninvasive model to generate the nomogram, the C-index was larger (**Table 2**). The final model for individualized assessment with the incorporated risk factors was depicted as a nomogram (**Figure 2**).

**Figure 3:**
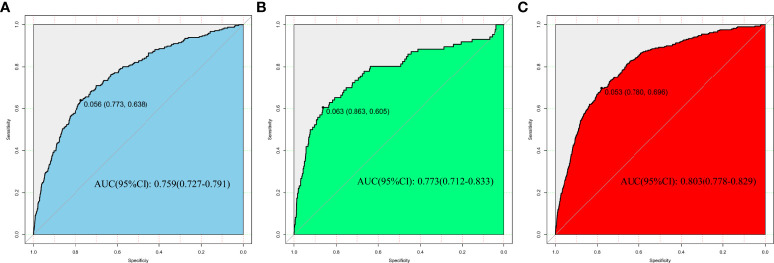
Nomogram performance for assessing the rate of isolated high 2-hour plasma glucose. **(A)** Development dataset. **(B)** Internal validation dataset. **(C)** External validation dataset.

In the development, interval validation, and external validation datasets, a ROC curve was created to test the risk variables’ capacity to identify the incidence of isolated high 2-hour plasma glucose (**Figure 3**). The AUCs of the ROC were 0.759 (95 percent CI: 0.727-0.791), 0.781 (95 percent CI: 0.712-0.833), and 0.803 (95 percent CI: 0.778-0.829) in the development, internal validation, and external validation datasets, respectively.

**Figure 4:**
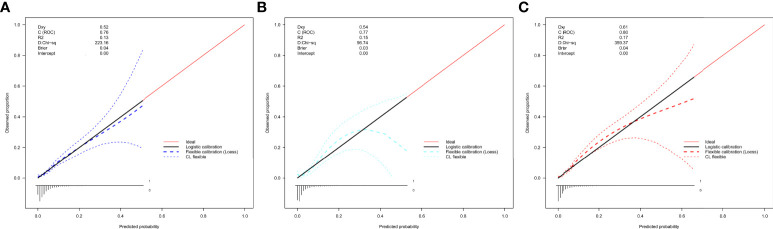
Calibration curves of the nomogram for rates of isolated high 2-hour plasma glucose in the validation datasets. **(A)** Development datasets (Guangzhou). **(B)** Internal validation datasets (Guangzhou). **(C)** External validation datasets (Changchun). The curves represent calibration of each model in terms of agreement between the assessed risk and observed outcomes of isolated.

In the internal and external validation datasets, the calibration curve of the evaluating nomogram for the prevalence of isolated high 2-hour plasma glucose demonstrated good agreement (**Figure 4**). The Development datasets (Guangzhou) (P = 0.600), and external validation datasets (Changchun) (P = 0.825) produced nonsignificant P-values for the Hosmer-Lemeshow test, confirming satisfactory nomogram model calibration.

### Clinical use

**Figure 5:**
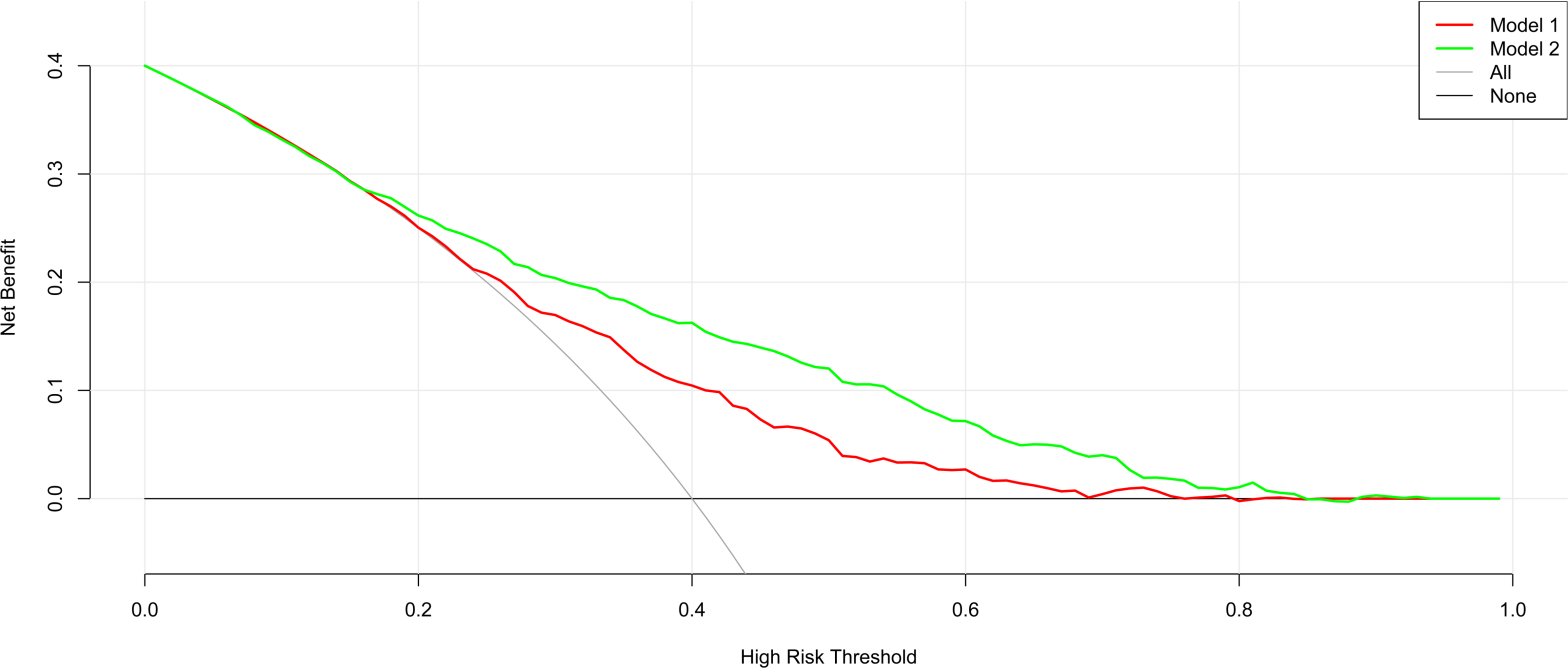
Decision curve analysis for the nomogram model in the development and internal and external validation datasets.

The nomogram’s clinical usefulness was examined using a decision curve analysis (**Figure 5**). The standard net benefit utilizing the models was represented on the y-axis, and the threshold probability for isolated high 2-hour plasma glucose was plotted on the x-axis. The decision curve revealed that the nomogram model was more effective for determining the prevalence of isolated high 2-hour plasma glucose. than either the treat-all-participant scheme or the treat-none scheme when the threshold probability was between 0.2 and 0.7. In both the development and validation datasets, the net effect was positive within this range.

## Discussion

In this study, a nomogram model was developed and validated for assessing the risk of isolated high 2-hour plasma glucose. The analysis was based on 2 large cross-sectional datasets from Guangzhou (South China) and Changchun (North China). The model incorporates 10 indices: gender, age, drinking status, marriage status, history of hypertension, history of hyperlipidemia, SBP, WHR, FPG, and HbA1c. Good discrimination was demonstrated in both the development and validation sets of subjects. The performance of the nomogram model is adequate for detecting isolated high 2-hour plasma glucose in the Chinese population.

Relying on the 2-h OGTT for the prevention and diagnosis of DM has several advantages. It can establish whether a subject has impaired glucose tolerance or undiagnosed type 2 diabetes, predicts the risk of heart disease more effectively than FPG, and possibly diagnoses more patients with diabetes (15, 22, 23). Besides, due to the lack of standardized detection methods and the detection results are susceptible to many factors, such as detection methods, anemia and abnormal hemoglobin diseases, red blood cell conversion speed, age and so on, the use of HbA1c alone as the diagnostic as a diagnostic criterion for diabetes has a low predictive value (24). However, the American Diabetes Association suggests abandoning the 2-h OGTT because it is time consuming, poorly reproducible, and not well accepted by patients (25). Furthermore, the 2-h OGTT is often accompanied by adverse reactions such as nausea and vomiting (26). Thus, if subjects who do not need 2-h OGTT could be identified, this would save medical resources and reduce the rate of adverse reactions. In this study, we developed and validated a nomogram model for assessing the risk of high 2-hour plasma glucose. The nomogram is valuable for distinguishing subjects who need a 2-h OGTT, and thereby improves the management of patients.

The development dataset was obtained from Guangzhou from South China. Through univariate analysis and subsequent multivariable analysis, the following independent factors were identified: gender, age, drinking status, marriage status, history of hypertension, history of hyperlipidemia, SBP, WHR, FPG and HbA1c.

To minimize over-fitting and assess generalizability, the nomogram must be validated. Calibration plots demonstrated the best agreement between prediction and actual observation in the current investigation, ensuring the repeatability and dependability of the constructed nomogram. The model also suited the datasets, which included patients from both the south (internal validation) and north (external validation) of China. This supports the widespread usage of this nomogram, independent of area, lifestyle, or health-care inequities. To our best knowledge, this is the first nomogram for identifying participants who are risk of isolated high 2-hour plasma glucose. It is based on a large database from South and North China. Using an easy scoring system, both physicians and patients can determine if a 2-h OGTT is necessary, even when both the FPG and HbA1c levels have not reached the criteria for a diagnosis of DM. Identifying subgroups of participants at varied risk of isolated high 2-hour plasma glucose can benefit the selection of care options. Determining which subjects require additional 2-h OGTT for the diagnosis of DM remains controversial. This scoring system should help physicians address such issues.

Because the nomogram based only on noninvasive factors may predict high 2-hour plasma glucose, it has wide application for individuals in various medical settings, including those with limited resources. The semi-lab model is more appropriate for individuals who are under the care of community doctors, and is also useful for epidemiologic studies for isolated high 2-hour plasma glucose screening.

## Limitations

There are several drawbacks to this study. First, the research population was mostly female, owing to the fact that we only invited residents above the age of 40, and women make up the majority of the population in this age range. Second, because the nomogram model only included Chinese individuals, it may not be typical of other ethnic groups, particularly those in foreign nations. The model’s strength, to some part, stems from the fact that it was built using data from a large national representative sample in South China and verified using data from an external population in North China. Data that have been included in other published nomogram models concerning daily consumption of vegetables, fruits, or berries (27) and use of steroids (28) were not available in our study questionnaire. It is not clear to what extent these missing variables will affect the need to assess risk of isolated high 2-hour plasma glucose. In addition, although using a semi-lab nomogram model to screen isolated high 2-hour plasma glucose may reduce the number of individuals who undergo testing, the lack of testing might miss people with DM. The choice of cutoff value with its related sensitivity and specificity should depend on the purpose of the semi-lab nomogram model. Besides, this study only explored the diagnostic threshold of diabetes, failing to provide the diagnostic critical value of impaired glucose tolerance, which requires further research. Last but not least, incomplete data compilation may influence the interpretation of the result of this study. Therefore, other important characteristics and lifestyle information, such as regional dietary habits and economic income, should also be considered to strengthen the findings of the study.

## Conclusion

In summary, we initially established and validated a novel nomogram based on two large databases for assessing patients with risk of isolated high 2-hour plasma glucose. With this model, clinicians may more precisely identify those individuals who are in need of a 2-h OGTT test for diagnosis of DM, when both FPG and HbA1c levels are normal. This nomogram should optimize screening of DM in these patients.

## Data availability statement

The original contributions presented in the study are included in the article/supplementary material. Further inquiries can be directed to the corresponding authors.

## Ethics statement

The studies involving human participants were reviewed and approved by the Institutional Review Board of the Sun Yat-sen Memorial Hospital affiliated with Sun Yat-sen University. The patients/participants provided their written informed consent to participate in this study.

## Author contributions

Conceiving ideas and experiment design: KS, LYa and GuW.

Actual experimentation: XX, LYo, XH, DL, YL, CH, FL, CS, CC, JL, YQ, CW, YaL, MX, MR, CY. Manuscript writing: KS, XX, and LYo. All authors contributed to the article and approved the submitted version.

## Funding

This work was supported by grants from: 1) National Key Research and Development Project of China (2016YFC0901204); 2) The National Science Foundation of China (81970696); 3) The Natural Science Foundation of China (82000784); 4) Sun Yat-sen Clinical Research Cultivating Program (SYS-Q-201801); 5) Sun Yat-sen University Clinical Research 5010 Program (2018021); 6) Medical Science and Technology Research Fund Project of Guangdong Province (A2019391); 7) Science and Technology Planning Project of Guangdong Province, China (2014A020212069); 8) Guang Dong Clinical Research Center for Metabolic Diseases (2020B1111170009); 9) Natural Science Foundation of Guangdong Province, China (2022A1515012111); 10) Guangzhou Basic Research Program [Basic and Applied Basic Research Project] (202102080101).

## Acknowledgments

We are indebted to the participants in the present study for their outstanding support and to our colleagues for their valuable assistance. The authors acknowledge Guang Ning (RuiJin Hospital, Shanghai Jiao Tong University School of Medicine, Shanghai, China) for valuable advice regarding data collection.

## Conflict of interest

The authors declare that the research was conducted in the absence of any commercial or financial relationships that could be construed as a potential conflict of interest.

## Publisher’s note

All claims expressed in this article are solely those of the authors and do not necessarily represent those of their affiliated organizations, or those of the publisher, the editors and the reviewers. Any product that may be evaluated in this article, or claim that may be made by its manufacturer, is not guaranteed or endorsed by the publisher.
